# A nomogram for predicting mortality of patients initially diagnosed with primary pulmonary tuberculosis in Hunan province, China: a retrospective study

**DOI:** 10.3389/fcimb.2023.1179369

**Published:** 2023-06-02

**Authors:** Dan Li, Si-Yuan Tang, Sheng Lei, He-Bin Xie, Lin-Qi Li

**Affiliations:** ^1^ Xiangya School of Nursing, Central South University, Changsha, Hunan, China; ^2^ College of Applied Technology, Hunan Open University, Changsha, Hunan, China; ^3^ Interventional Radiology Center, Hunan Chest Hospital, Changsha, Hunan, China; ^4^ Department of Drug Clinical Trial Institutions, The Affiliated Changsha Central Hospital, Hengyang Medical School, University of South China, Changsha, Hunan, China; ^5^ School of Public Health, University of South China, Hengyang, China

**Keywords:** initially diagnosed, primary pulmonary tuberculosis, mortality, prognostic, nomogram

## Abstract

**Objective:**

According to the Global Tuberculosis Report for three consecutive years, tuberculosis (TB) is the second leading infectious killer. Primary pulmonary tuberculosis (PTB) leads to the highest mortality among TB diseases. Regretfully, no previous studies targeted the PTB of a specific type or in a specific course, so models established in previous studies cannot be accurately feasible for clinical treatments. This study aimed to construct a nomogram prognostic model to quickly recognize death-related risk factors in patients initially diagnosed with PTB to intervene and treat high-risk patients as early as possible in the clinic to reduce mortality.

**Methods:**

We retrospectively analyzed the clinical data of 1,809 in-hospital patients initially diagnosed with primary PTB at Hunan Chest Hospital from January 1, 2019, to December 31, 2019. Binary logistic regression analysis was used to identify the risk factors. A nomogram prognostic model for mortality prediction was constructed using R software and was validated using a validation set.

**Results:**

Univariate and multivariate logistic regression analyses revealed that drinking, hepatitis B virus (HBV), body mass index (BMI), age, albumin (ALB), and hemoglobin (Hb) were six independent predictors of death in in-hospital patients initially diagnosed with primary PTB. Based on these predictors, a nomogram prognostic model was established with high prediction accuracy, of which the area under the curve (AUC) was 0.881 (95% confidence interval [Cl]: 0.777-0.847), the sensitivity was 84.7%, and the specificity was 77.7%.Internal and external validations confirmed that the constructed model fit the real situation well.

**Conclusion:**

The constructed nomogram prognostic model can recognize risk factors and accurately predict the mortality of patients initially diagnosed with primary PTB. This is expected to guide early clinical intervention and treatment for high-risk patients.

## 1 Introduction

According to the Global Tuberculosis Report for three consecutive years, tuberculosis (TB) is the second leading infectious killer after COVID-19, with higher mortality than human immunodeficiency virus (HIV)/acquired immunodeficiency syndrome (AIDS). It is also noted that 2022 is the first year when TB incidence and death rates have increased. Impacted by COVID-19 pandemic, the effects of TB prevention and treatment can be interrupted or changed over the years (Dheda et al., 2022). In 2021, China ranked third worldwide, with TB incidence (new cases per 100,000 population) estimated at 55 and TB mortality at 4%. Moreover, in 2021, there were 780,000 new TB cases in China, of which new pulmonary tuberculosis (PTB) cases accounted for 95%. These results indicate the high TB burden in China. In the new era of COVID-19 pandemic, China has faced huge challenges in TB prevention and treatment. Recently, high-sensitivity tools have been introduced to screen out, intervene, and treat TB to reduce TB mortality (Chakaya et al., 2022).

The lungs are the most common site of TB infection, and PTB leads to the highest mortality among TB diseases. Patients with different types of PTB or with different courses of PTB will differ in various aspects, such as clinical symptoms, hematologic manifestations, treatment methods, intervention methods, and even treatment outcomes (Sauer et al., 2018; Mohidem et al., 2021; Kheirandish et al., 2022). Regretfully, in previous TB prognostic studies, the research objects were classified mainly based on: 1) drug resistance; 2) HIV status; 3) comorbidities; 4) PTB or extrapulmonary TB (EPTB) (Jamal et al., 2020; Liu et al., 2020; Li et al., 2022). No previous studies targeted the PTB of a specific type or in a specific course, so models established in previous studies cannot be accurately feasible for clinical treatments.

This study aimed to construct a nomogram prognostic model to identify the risk factors for in-hospital patients initially diagnosed with primary PTB to offer the most effective therapeutic schemes, the most appropriate case management, and the optimal resource allocation for patients who are the least likely to be cured but most likely to benefit from the intervention measures. The study breaks through the limitations of the same type of study in terms of the lack of segmentation of the target population and the lack of clinical usefulness of the selection of predictors. To precisely move the intervention study population forward, patients with a primary diagnosis of tuberculosis were selected, and statistically significant risk factors for mortality were screened. A prognostic intervention model for death was then constructed, which was suitable for clinical applications. This study’s findings have three significant advantages. First, primary TB patients account for 95% of new TB cases annually, and the model is highly targeted and applicable to a wide range of targets. Second, risk predictors of mortality prognosis are easily accessible and identifiable in the clinical setting. Third, internal model validation was performed; the model fit and predictive value were better, and the prediction method was simple and fast.

## 2 Research objects and methods

### 2.1 Research objects

The retrospective case-control method included 1,809 in-hospital patients initially diagnosed with primary PTB at Hunan Chest Hospital from January 1, 2019, to December 31, 2019. In China, patients are received and treated at designated points and can obtain partial subsidies from health insurance (Liu et al., 2022b). Hunan Chest Hospital is one of the provincial-level designated points for TB treatment in China, which has received and treated the most TB patients in Hunan province. Therefore, TB cases in Hunan Chest Hospital reflect the epidemiological trends and disease characteristics of TB throughout Hunan province.

The research objects were included based on the following criteria. 1) The patients were diagnosed with primary PTB based on the Health Industry Standard of the People’s Republic of China—Diagnosis for pulmonary tuberculosis (WS 288-2017) (China, N.H.a.F.P.C.o.t.P.s.R.o, 2017). 2) The patients never took anti-TB drugs or received irregular chemotherapy within one month after being diagnosed with primary PTB (Cao et al., 2007). 3) During the hospital stay and after discharge, the patients received standardized chemotherapy for initially diagnosed primary PTB according to the recommendations of WHO and Technical Specifications for TB Prevention and Control in China (Zhang et al., 2014). Two months of intensive treatment with isonicotinic hydrazine, rifepine, pyrazinamide, ethambutanol sterilization combined with bacteriostatic drugs, followed by four months of consolidation treatment with isonicotinic hydrazine and rifepine. 4) The patients were equal to or older than 18 years old with a hospital stay of more than three days. 5) Before a hospitalization, the patients never took cortin or drugs that affected the lab-tested albumin (ALB), lymphocyte count, and other indicators for the long term. 6) The patients were not pregnant or lactating women. 7) The patients were not severe with HIV/AIDS, benign or malignant tumors, organ failure, or other diseases. 8) The clinical baseline data of patients were complete.

The study was conducted in accordance with the Declaration of Helsinki. As this study was based on retrospective research of patient data from a case management system, with visa-free informed consent for ethical approvals, and oral knowledge with respondents during telephone surveys with respondents. This visa-free informed consent for ethical approvals was approved by the Nursing and Behavioral Medicine Research Ethics Review Committee, Xiangya Nursing School of Central South University(ID:E2022104).

### 2.2 Data collection

This study collected data on in-hospital PTB patients from the case management system in Hunan Chest Hospital, including general demographic data (gender, age, marital status, type of health insurance, smoking history, drinking history, and dust exposure history), in-hospital comorbidity data [hypertension, diabetes, chronic gastritis, and coronary heart disease (CHD]), clinic-related data, and experimental parameters [height, weight, ALB, lymphocyte count, creatinine, cholinesterase (CHE), total cholesterol, C-reactive protein (CRP), hemoglobin (Hb), and platelet (PLT]). The above data are from the first week of admission of patients initially diagnosed with primary PTB. Smoking no less than 20 cigarettes per week was considered a smoking history. Drinking no less than five times or 500 mL weekly was considered to have a drinking history. Exposure to an extremely dusty environment of no less than five times was considered to have a dust exposure history. In-hospital comorbidity data were obtained from the chief complaints of patients, which is consistent with the review results after admission to the hospital. For reflecting the nutritional status of research objects, this study converted the height and weight into the body mass index (BMI) according to *Medical Nutrition Treatment of Overweight/Obesity in China (2021)*, where BMI values less than 18.5 kg/m² are considered underweight, BMI values from 18.5 to 24.99 kg/m² are normal weight. BMI values > 25 kg/m² were overweight or obese (Chen et al., 2004; Sahile et al., 2021).

This study obtained follow-up data from the research subjects and understood patient mortality on a phone visit from September 1, 2022, to September 31, 2022. The outcomes were collected from the first hospitalized treatment upon initial diagnosis of primary PTB to the end of the phone visit, the average duration of which was three years. According to WHO classification standards, this study classified the treatment results of research subjects into non-survivor group (died) and survivor group (cured, drug-resistant, and relapsing) (Eurosurveillance Editorial Team, 2013). Survivors further returned to the study to see if tuberculosis drug resistance, and recurrence of tuberculosis during the treatment period.

### 2.3 Statistical analysis

R software V4.2.2 (http://www.R-project) was used for data input and statistical analysis. Continuous variables were normally distributed and described as mean and standard deviation (SD), whereas categorical variables were expressed as frequency percentages. The confidence interval (Cl) was 95% (α= 0.05), and *p < 0.05* (bilateral) is statistically significant. The *t*-test was conducted to compare the continuous variables with the research results, and *chi-square (χ²)* test was adopted to compare the categorized variables with research results. Binary logistic regression was used for univariate analysis, and backward stepwise logistic regression was used for multivariate analysis to screen for statistically significant prognostic risk factors for patients initially diagnosed with primary PTB. R software was used to construct the nomogram prediction model based on independent risk factors in the training set and to validate the constructed model using the validation set.

## 3 Results

### 3.1 Characteristics of research objects

**Figure 1:**
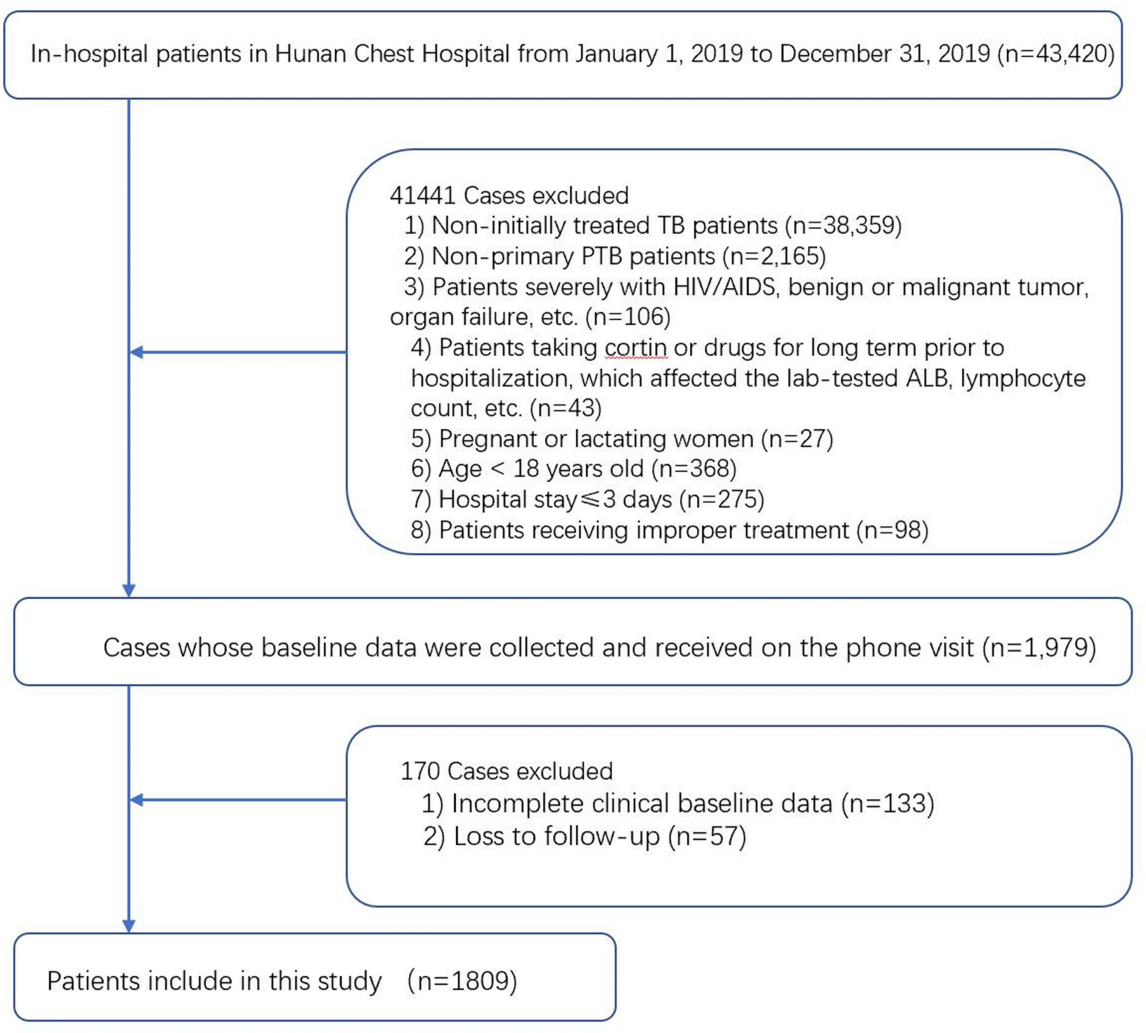
Study design. A total of 1,809 in-hospital patients initially dragonized with primary PTB with complete relevant data were enrolled in this study.

This study included 1,809 in-hospital patients initially diagnosed with primary PTB who were screened, as presented in **Figure 1**. The research objects were randomly split into a training set (n=1449) and a validation set (n=360) at a ratio of 8:2 and a random seed of 1314.

Among 1,809 research subjects, 83 patients (4.6%) died within three years of treatment, including 72 patients (4.9%) in the training set and 11 patients (3.1%) in the validation set. 174 patients(9.6%) developed tuberculosis resistance during the three-year follow-up period, including 131(9.0%) in the training set and 43(11.9%) in the validation set. 389 (21.5%)patients experienced tuberculosis recurrence during the three-year follow-up period, including 305(21.0%) in the training set and 84(23.3%) in the validation set.

In this study, 22 variables were analyzed, the results of which were as follows. 1) The average age of the research subjects was 48 years. 2) The number of male patients (64%) was higher than that of female patients (37%). 3) Smokers (65.2%) and drinkers (76.2%) accounted for relatively high proportions. 4) Patients with comorbidities accounted for 25.5%, including 8.9% hypertension, 10.6% diabetes, 2.9% HBV, 5.7% chronic gastritis, and 5.7% CHD. 5) Bachelordom represented 24.6% of the participants. 6) Patients without health insurance accounted for 13.9% of the patients. 7) Underweight patients accounted for 24.5%, and overweight patients accounted for 9.3%, for details about the demographic variables and clinical characteristics of the research subjects (**Table 1**).

**Table 1:** Baseline characteristics of included in-hospital patients initially dragonized with primary PTB.

Variable	Total cohort (n=1809)	Training set (n=1449)	Validation set (n=360)
	number (percentage)	number (percentage)	number (percentage)
Treatment outcomes			
Survivors	1726 (95.4%)	1377 (95.1%)	348 (96.9%)
Non-survivors	83 (4.6%)	72 (4.9%)	12 (3.1%)
Drug-resistant TB(DRTB)			
No	1635(90.4%)	1318(91.0%)	317(88.1%)
Yes	174(9.6%)	131(9.0%)	43(11.9%)
Relapse			
No	1420(78.5%)	1144(79.0%)	276(76.7%)
Yes	389(21.5%)	305(21.0%)	84(23.3%)
Gender			
Male	1157 (64.0%)	942 (65.0%)	215 (59.7%)
Female	652 (36.0%)	507 (35.0%)	145 (40.3%)
Smoking			
No	1179 (65.2%)	947 (65.4%)	232 (64.4%)
Yes	630 (34.8%)	502 (34.6%)	128 (35.6%)
Drinking			
No	1378 (76.2%)	1107 (76.4%)	271 (75.3%)
Yes	431 (23.8%)	342 (23.6%)	89 (24.7%)
Dust exposure			
No	1688(93.3%)	1358 (93.7%)	33 (91.7%)
Yes	121 (6.7%)	91 (6.3%)	30 (8.3%)
Hypertension			
No	1648(91.1%)	1319 (91.0%)	329 (91.4%)
Yes	161 (8.9%)	130 (9.0%)	31 (8.6%)
Diabetes			
No	1618 (89.4%)	1304 (90.0%)	314 (87.2%)
Yes	191 (10.6%)	145 (10.0%)	46 (12.8%)
Hepatitis B virus (HBV)			
No	1756 (97.1%)	1406 (97.0%)	350 (97.2%)
Yes	53 (2.9%)	43 (3.0%)	10 (2.8%)
Gastritis			
No	1706 (94.3%)	1369 (94.5%)	337 (93.6%)
Yes	103 (5.7%)	80 (5.5%)	23 (6.4%)
Coronary heart disease (CHD)			
No	1706 (94.3%)	1369 (94.5%)	337 (93.6%)
Yes	103 (5.7%)	80 (5.5%)	23 (6.4%)
Comorbidity			
No	1348 (74.5%)	1087 (75.0%)	261 (72.5%)
Yes	461 (25.5%)	362 (25.0%)	99 (27.5%)
Bachelordom			
No	445 (24.6%)	361 (24.9%)	84 (23.3%)
Yes	1364 (75.4%)	1088 (75.1%)	276 (76.7%)
Health insurance			
No	251 (13.9%)	199 (13.7%)	52 (14.4%)
Yes	1558 (86.1%)	1250 (86.3%)	308 (85.6%)
BMI (kg/m^2^)			
Normal	1196 (66.1%)	937 (64.7%)	259 (71.9%)
Underweight	444 (24.5%)	372 (25.7%)	72 (20%)
Overweight	169 (9.3%)	140 (9.6%)	29 (8.1%)
**Age**, years	47.59 ± 17.65	47.44 ± 17.62	48.18 ± 16.55
**Albumin** (ALB) (g/L)	40.90 ± 5.61	40.95 ± 5.55	40.74 ± 5.22
**Creatinine** (Cr) (μmol/L)	69.26 ± 25.31	69.43 ± 24.93	68.60 ± 25.16
**Cholinesterase** (CHE) (U/L)	7964.37 ± 2453.63	7960.79 ± 2426.87	7981.94 ± 2479.55
**Total cholesterol** (TC) (mmol/L)	4.29 ± 1.02	4.28 ± 1.00	4.32 ± 0.99
**C-reactive protein** (CRP) (mg/L)	25.99 ± 43.04	25.85 ± 43.78	26.56 ± 38.18
**Lymphocyte count** (Lym) (*10^9^/L)	1.60 ± 0.66	1.59 ± 0.67	1.63 ± 0.65
**Hemoglobin** (Hb) (g/L)	122.32 ± 17.66	122.40 ± 17.42	122.20 ± 18.14
**Platelet count** (PLT) (*10^9^/L)	253.73 ± 91.58	255.70 ± 91.40	245.80 ± 88.97

Univariate binary logistic regression analysis was performed for 1,448 cases in the training set. The results demonstrated that 13 variables, including sex, smoking, drinking, hypertension, HBV, comorbidity, BMI, age, ALB, CHE, CRP, lymphocyte count, and Hb, were statistically different between the survivor and non-survivor group (*p<0.05*), as illustrated in **Table 2**. Univariate andmultivariate logistic regression analysis of the training set of the population of in-hospital patients initially dragonized with primary PTB was shown in **Table 3**.

**Table 2:** Demographics and clinical characteristics of the population of in-hospital patients initially dragonized with primary PTB.

Variable	Total (n=1449)	Survivors (n=1377)	Non-survivors (n=72)	P value
		number (percentage)	number (percentage)	
Drug-resistant TB(DRTB)				
No	1318	1253 (91%)	65 (90.3%)	1.000
Yes	131	124 (9%)	7 (9.7%)	
Relapse				
No	1144	1088 (79%)	56 (77.8%)	.919
Yes	305	289 (21%)	16 (22.2%)	
Gender				
Male	942	884 (64.2%)	58 (80.6%)	0.007
Female	507	493 (35.8%)	14 (19.4%)	
Smoking				
No	947	914 (66.4%)	33 (45.8%)	<0.001
Yes	502	463 (33.6%)	39 (54.2%)	
Drinking				
No	1107	1065 (77.3%)	42 (58.3%)	<0.001
Yes	342	312 (22.7%)	30 (41.7%)	
Dust exposure				
No	1358	1291 (93.8%)	67 (93.1%)	1.000
Yes	91	86 (6.2%)	5 (6.9%)	
Hypertension				
No	1319	1259 (91.4%)	60 (83.3%)	0.033
Yes	130	118 (8.6%)	12 (16.7%)	
Diabetes				
No	1304	1244 (90.3%)	60 (83.3%)	0.084
Yes	145	133 (9.7%)	12 (16.7%)	
Hepatitis B virus (HBV)				
No	1406	1340 (97.3%)	66 (91.7%)	0.017
Yes	43	37 (2.7%)	6 (8.3%)	
Gastritis				
No	1369	1300 (94.4%)	69 (95.8%)	0.801
Yes	80	77 (5.6%)	3 (4.2%)	
Coronary heart disease (CHD)				
No	1369	1300 (94.4%)	69 (95.8%)	0.801
Yes	80	77 (5.6%)	3 (4.2%)	
Comorbidity				
No	1087	1045 (75.9%)	42 (58.3%)	0.001
Yes	362	332 (24.1%)	30 (41.7%)	
Bachelordom				
No	361	348 (25.3%)	13 (18.1%)	0.215
Yes	1088	1029 (74.7%)	59 (81.9%)	
Health insurance				
No	199	185 (13.4%)	14 (19.4%)	0.205
Yes	1250	1192 (86.6%)	58 (80.6%)	
BMI (kg/m^2^)				
Normal	937	905 (65.7%)	32 (44.4%)	<.001
Underweight	372	337 (24.5%)	35 (48.6%)	
Overweight	140	135 (9.8%)	5 (6.9%)	
**Age**, years	47.4 ± 17.6	46.4 ± 17.3	67.2 ± 12.5	<.001
**Albumin** (ALB) (g/L)	40.9 ± 5.6	41.3 ± 5.4	35.0 ± 5.4	<.001
**Creatinine** (Cr) (μmol/L)	69.4 ± 24.9	69.2 ± 24.1	73.4 ± 32.0	0.273
**Cholinesterase** (CHE) (U/L)	7960.8 ± 2426.9	8066.1 ± 2422.0	5933.6 ± 2745.4	<.001
**Total cholesterol** (TC) (mmol/L)	4.3 ± 1.0	4.3 ± 1.0	4.2 ± 1.4	0.438
**C-reactive protein** (CRP) (mg/L)	25.9 ± 43.8	24.9 ± 41.8	43.4 ± 51.6	0.004
**Lymphocyte count** (Lym) (*10^9^/L)	1.6 ± 0.7	1.6 ± 0.6	1.3 ± 0.8	0.002
**Hemoglobin** (Hb) (g/L)	122.4 ± 17.4	123.1 ± 17.3	108.1 ± 17.2	<.001
**Platelet count** (PLT) (*10^9^/L)	255.7 ± 91.4	254.4 ± 91.2	280.2 ± 126.8	0.093

**Table 3:** Univariate and multivariate logistic regression analysis of the training set of the population of in-hospital patients initially dragonized with primary PTB.

Variable	Univariate analysis			Multivariate analysis		
	OR	95%CI	P	OR	95%CI	P
**DRTB**	1.09	0.49-2.42	.836			
**Gender**	2.31	1.28-4.18	0.01			
**Smoking**	2.33	1.45-3.76	<0.001	1.56	0.85-2.85	0.15
**Drinking**	2.44	1.50-3.96	<0.001	1.97	1.08-3.60	0.03
**Hypertension**	2.13	1.12-4.08	0.02			
**Hepatitis B virus** (HBV)	3.29	1.34-8.08	0.01	3.58	1.23-10.41	0.02
**Comorbidity**	2.25	1.38-3.65	0.00			
BMI (kg/m^2^)						
Normal						
Underweight	2.94	1.79-4.82	<0.001	1.79	1.01-3.16	0.04
Overweight	1.05	0.40-2.73	0.93	1.96	0.71-5.44	0.20
**Age**, years	1.10	1.07-1.12	<0.001	1.08	1.06-1.11	<0.001
**Albumin** (ALB) (g/L)	0.81	0.78-0.85	<0.001	0.90	0.84-0.96	0.00
**Cholinesterase** (CHE) (U/L)	1.00	1.00-1.00	<0.001			
**C-reactive protein** (CRP) (mg/L)	1.01	1.00-1.01	<0.001	1.00	0.99-1.00	0.16
**Lymphocyte count** (Lym) (*10^9^/L)	0.42	0.27-0.66	<0.001			
**Hemoglobin** (Hb) (g/L)	0.96	0.94-0.97	<0.001	0.98	0.96-1.00	0.04

CI, confidence interval; OR, odds ratio; p < 0.05.

### 3.2 Risk predictors for in-hospital patients initially diagnosed with primary PTB

**Figure 2:**
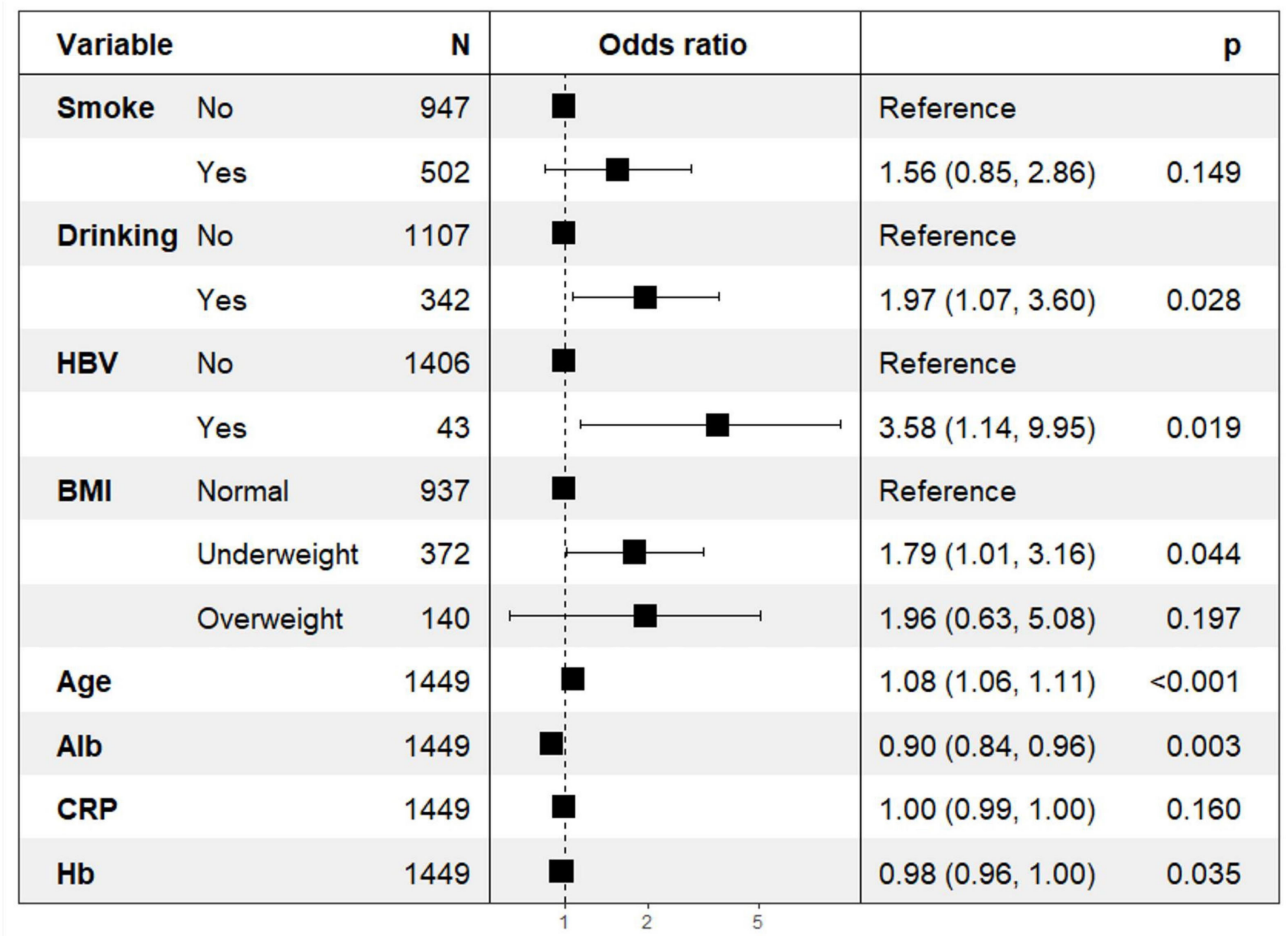
Backward stepwise logistic regression analysis of the training set of the population of in-hospital patients initially dragonized with primary PTB.

Statistically significant variables were included in multivariate binary logistic regression analysis. The results in **Figure 2** indicate that drinking (*p*=0.03; OR=1.97; 95% CI: 1.07–3.60), HBV (*p*=0.02; OR=3.58; 95% CI: 1.14–9.95), BMI (*p*=0.04; OR=1.79; 95% CI: 1.01–3.16), age (*p*<0.001; OR=1.08; 95% CI: 1.06–1.11), ALB (*p*=0.003; OR=0.90; 95% CI: 0.84–0.96), and Hb (*p*=0.035; OR=0.98; 95% CI: 0.96–1.00) were independent risk predictors for death (*p*<0.05) in the prognosis of in-hospital patients initially diagnosed with primary PTB (**Figure 2**).

**Figure 3:**
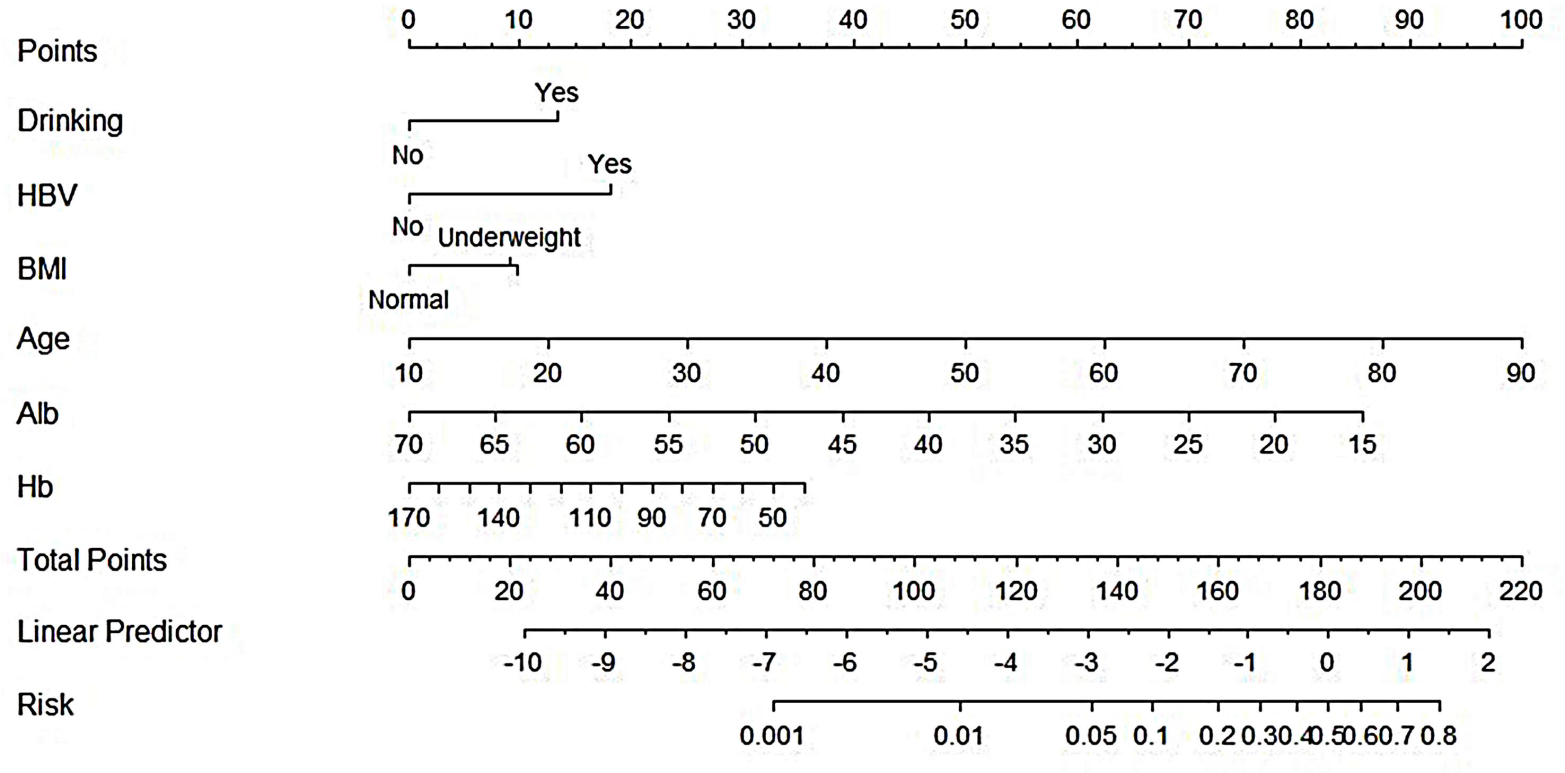
Nomogram to predict the outcomes of patients initially diagnosed with primary PTB.

With drinking, HBV, BMI, age, ALB, and Hb included in the prognosis of in-hospital patients initially diagnosed with primary PTB as independent risk predictors for death, R software established the nomogram prognostic model for mortality prediction to obtain the points corresponding to every predictor. The sum of these points was considered the death probability of in-hospital patients initially diagnosed with primary PTB, as described in **Figure 3**.

The nomogram model was adopted to obtain the points corresponding to every predictor and then calculate the total points considered as the death probability of in-hospital patients initially diagnosed with primary PTB. The total number of points is then calculated.

**Figure 4:**
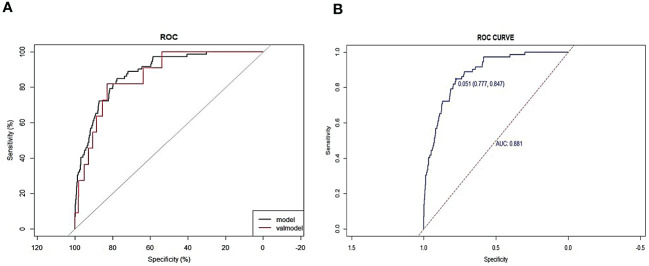
Calibration curves of the nomogram in the training set **(A)** and validation set **(B)**.

**Figure 5:**
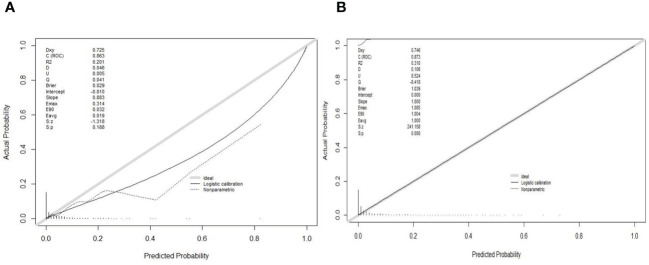
Calibration of the nomogram to predict the death in the training set **(A)** and validation set **(B)**.

**Figure 6:**
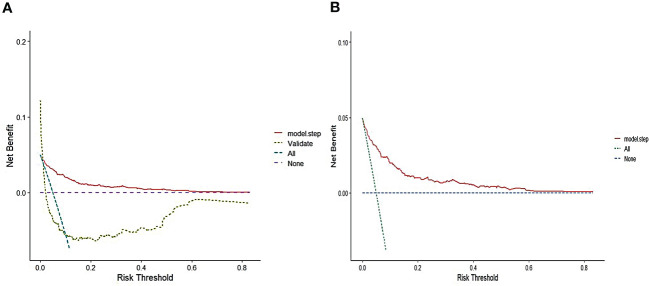
Decision cure analysis(DCA) for the nomogram in the training set **(A)** and validation set **(B)**.

The area under the receiver operating characteristic (ROC) curve (AUC) was used to evaluate the predictive accuracy of the constructed nomogram model. In the training set, AUC of the model was 0.881, with a sensitivity of 84.7% and specificity of 77.7% (**Figure 4**). In the validation set, AUC of the model was 0.907 (**Figure 4**). Consequently, the internal and external validation results were relatively consistent (**Figure 4**). R software was adopted to establish the calibration curves (1,000 bootstrapping samples) of the training set (**Figure 5A**) and the validation set (**Figure 5B**), where X-axis represents the predicted death probability of in-hospital patients initially diagnosed with primary PTB and Y-axis represents the observed death probability of in-hospital patients initially diagnosed with primary PTB. The calibration curves determined that the model effectively fitted the actual situation and had a high predictive value. Moreover, R software was used to draw the clinical decision curves for the training set (**Figure 6A**) and validation set (**Figure 6B**), the area of which was greater than 0, indicating that the model had high clinical effectiveness.

## 4 Discussion

Mycobacterium tuberculosis (MTB) infection is a dynamic process in which the systemic immune response is passively activated. The damaging immune and inflammatory responses of human tissues are subject to the infection site, infection cycle, and bacterial aggregation (Natarajan et al., 2020). Based on the *Health Industry Standard of the People’s Republic of China—Classification of Tuberculosis (WS 196-2017)*, PTB is categorized into primary PTB, hematogenous PTB, secondary PTB, tuberculous pleurisy, and EPTB. From the perspective of histology, the organs of patients with any of the five types of PTB generate granulomatous inflammation (Acharya et al., 2020). However, because of different immune and inflammatory responses, there will be a significant difference in aspects such as imagology, clinical course, test indicators, and therapeutic scheme (Hunter, 2018; Cohen et al., 2022). Previous studies generally selected a wide range of research objects to establish TB-related prediction models, especially prognosis prediction models, reducing the practicability of these models in the clinic (Tangri and Kent, 2015; Peetluk et al., 2021; Bert-Dulanto et al., 2022; Kuan, 2022).

Numerous studies have confirmed that different types and courses of PTB have different immune mechanisms, treatment outcomes, outcome probabilities, and risk factors (Koo et al., 2020; Li et al., 2020; Ali et al., 2021; Peetluk et al., 2022; Williams et al., 2022; Zhao et al., 2022). Based on this, this study accurately selected the patients initially diagnosed with primary PTB as the research objects to construct the prediction model and selected the predictors by combining the sensitive indicators and commonly used clinical indicators from previous relevant studies. These predictors can be routinely acquired in the clinical setting. Therefore, the constructed nomogram prediction model is highly operable to quickly predict the outcomes of patients initially diagnosed with primary PTB for accurate and targeted intervention in the clinic.

The selection of a follow-up period was a peculiarity in this study because the survival cycle of PTB patients varies greatly from country to country. A previous study on 8,240 TB patients in Andhra Pradesh, southern India, demonstrated that the death frequency in the four-year follow-up period was even (Ramakrishnan et al., 2021). Another previous study on time-related death factors of the 604 TB patients in southwestern Ethiopia indicated that the average time to death was five months (Jabir et al., 2022). A survival analysis of TB deaths from the Tuberculosis Disease and Mortality Surveillance Information System in Zhejiang province, China, concluded that 71.1% of 283 deaths caused by TB occurred within three years of diagnosis and treatment (Liu et al., 2022a). The main reason for this difference is that TB is closely related to body nutrition as a type of immune disease, and individual nutritional status is closely related to individual economic and socioeconomic status (Muttamba et al., 2020; Guo et al., 2022; Sinha et al., 2022). Therefore, we discovered that the results of a prognostic model are different for TB patients in different regions. Therefore, accurately matching the characteristics of the clinical application population is the first step toward constructing a prognostic model. The regions of patients with TB and their influence on their survival cycle by region must be considered. Combining case studies on PTB patients in China and the estimated probability of TB death in China, this study found that over the 3-year follow-up period, 4.89% of patients who had been initially diagnosed with primary PTB died. This indicated that the selection of the follow-up period reduced the bias error and greatly increased the clinical applicability of the model.

Compared with the treatment outcome prediction model for adult patients with the same type of PTB, age and BMI are common predictive risk factors affecting treatment outcomes. On the one hand, older PTB patients have a higher death possibility than younger patients (Bert-Dulanto et al., 2022). According to the statistical data in PTB case reports (2006–2020) from the Tuberculosis Information Management System (TBIMS) in China, PTB incidence and mortality in China increased with age (Dong et al., 2022). On the other hand, undernutrition is a risk factor for immunodeficiency and an important risk factor for PTB incidence and adverse outcomes (Ma et al., 2022). BMI is the macro indicator of human nutritional status, and ALB and Hb are the biomarkers to reflect human nutritional status (Du et al., 2022). Therefore, BMI, ALB, and Hb are mutually the cause and effect, also reflected in TB patients in China (Kamruzzaman, 2021; Ashenafi et al., 2022; Jiang et al., 2022). Therefore, this study considered age, BMI, ALB, and Hb as the core and easy-to-quantify indicators in the prediction model. These are important monitoring indicators for quantifying the intervention effects in high-risk patients initially diagnosed with primary PTB.

In demographic characteristics, drinking and HBV infection are also important risk predictors for death in the prognosis of in-hospital patients initially diagnosed with primary PTB because chronic alcohol and HBV reduce the expression of immune proteins in infected patients, further decreasing their immune function (Wigger et al., 2022). HBV infection cycle and the amount or frequency of drinking are positively correlated with TB susceptibility, indicating that both drinking and HBV infection are high-risk factors for the death of TB patients (Imtiaz et al., 2017; Echazarreta et al., 2018; Kang et al., 2021; Chen et al., 2022). In the later stage of standard anti-TB chemotherapy, as prescribed, the anti-TB drugs such as isoniazid (INH), rifampicin (RIF), and pyrazinamide (PZA) have hepatotoxic side effects for patients initially diagnosed with primary PTB. Drinking and HBV infection can exacerbate liver damage and increase fatality (Khan et al., 2021; Chou et al., 2022). Accordingly, to reduce the prognostic mortality of patients initially diagnosed with primary PTB, it is necessary to conduct routine lifestyle surveys and HBV screening before treatment to facilitate early clinical recognition, adjustment of therapeutic schemes, and disease management, further improving anti-TB treatment and reducing death outcomes.

One limitation of this study was that the evidence level of this retrospective study was relatively inferior. Second, the clinically relevant data and experimental parameters adopted in this study are only part of the clinical data of the patients, and other meaningful data may not be included. Further studies are required to widen the scope of these variables.

## 5 Conclusion

The constructed nomogram prognostic model verified that drinking, HBV, BMI, age, ALB, and Hb were the six independent risk predictors of death in hospital patients with primary PTB who were initially diagnosed. Consequently, the six risk predictors of in-hospital patients initially diagnosed with primary PTB must be screened, recognized, and intervened as early as possible to reduce patient mortality.

## Data availability statement

The original contributions presented in the study are included in the article/**Supplementary Material**. Further inquiries can be directed to the corresponding author.

## Ethics statement

The studies involving human participants were reviewed and approved by The Ethics Committee of Xiangya School of Nursing, Central South University, approved this study. Written informed consent for participation was not required for this study in accordance with the national legislation and the institutional requirements.

## Author contributions

DL and ST contributed to conception and design of the study. SL and LL organized the database. DL and HX performed the statistical analysis. DL wrote the first draft of the manuscript. All authors contributed to manuscript revision, read, and approved the submitted version.
